# Monitoring of in-hospital cardiac arrest events with the focus on Automated External Defibrillators – a retrospective observational study

**DOI:** 10.1186/s13049-015-0170-7

**Published:** 2015-10-31

**Authors:** Thomas Wurmb, Tina Vollmer, Peter Sefrin, Martin Kraus, Oliver Happel, Christian Wunder, Andreas Steinisch, Norbert Roewer, Sebastian Maier

**Affiliations:** Department of Anaesthesiology, University Hospital of Wuerzburg, Oberduerrbacherstrasse 6, 97080 Wuerzburg, Germany; Department of Anaesthesiology, Hospital of Ludwigsburg, Posilipoststrasse 4, 71640 Ludwigsburg, Germany; Emergency Services, Wuerzburg, Germany; Department of Internal Medicine II, St. Elisabeth Hospital Straubing GmbH, St. Elisabeth-Strasse 23, 97315 Straubing, Germany

**Keywords:** In-hospital cardiac arrest, Automated External Defibrillators, Chest-Compression rate, Team-training, Cardio-pulmonary resuscitation

## Abstract

**Background:**

Patients with cardiac arrest have lower survival rates, when resuscitation performance is low. In In-hospital settings the first responders on scene are usually nursing staff without rhythm analysing skills. In such cases Automated External Defibrillators (AED) might help guiding resuscitation performance. At the Wuerzburg University Hospital (Germany) an AED-program was initiated in 2007.

Aim of the presented study was to monitor the impact of Automated External Defibrillators on the management of in-hospital cardiac arrest events.

**Methods:**

The data acquisition was part of a continuous quality improvement process of the Wuerzburg University Hospital. For analysing the CPR performance, the chest compression rate (CCR), compression depth (CCD), the no flow fraction (NFF), time interval from AED-activation to the first compression (TtC), the time interval from AED-activation to the first shock (TtS) and the post schock pause (TtCS) were determined by AED captured data. A questionnaire was completed by the first responders.

**Results:**

From 2010 to 2012 there were 359 emergency calls. From these 53 were cardiac arrests with an AED-application. Complete data were available in 46 cases. The TtC was 34 (32–52) seconds (median and IQR).The TtS was 30 (28–32) seconds (median and IQR) . The TtCS was 4 (3–6) seconds (median and IQR) . The CCD was 5.5 ± 1 cm while the CCR was 107 ± 11/min. The NFF was calculated as 41 %.

ROSC was achieved in 21 patients (45 %), 8 patients (17 %) died on scene and 17 patients (37 %) were transferred under ongoing CPR to an Intensive Care Unit (ICU).

**Conclusion:**

The TtS and TtC indicate that there is an AED-user dependent time loss. These time intervals can be markedly reduced, when the user is trained to interrupt the AED’s “chain of advices” by placing the electrode-paddles immediately on the patient’s thorax. At this time the AED switches directly to the analysing mode. Intensive training and adaption of the training contents is needed to optimize the handling of the AED in order to maximize its advantages and to minimize its disadvantages.

## Background

Outcome is worse in patients with cardiac arrest when resuscitation performance, choreography and adherence to guidelines are low [1, 2]. Important components of cardio-pulmonary resuscitation (CPR) are the team-level logistics, monitoring, feed-back and metrics of CPR-performance as well [1–4]. To be prepared for in-hospital cardiac arrests the availability of specialized medical emergency teams (MET) is necessary to provide Advanced Cardiac Life Support (ACLS) [5]. Nevertheless in most cases Basic Life Support (BLS) is performed by the first responders on scene. In hospitals these are usually nurses without rhythm recognition skills and full-scale defibrillators are usually unavailable on regular wards. In those settings Automated External Defibrillators (AEDs) are considered to improve the quality of CPR of the first responder team (FRT) and to shorten the time from onset of cardiac arrest to the first defibrillation in shockable rhythms [6, 7]. Besides the technical feature to analyse heart-rhythms and to deliver a shock where indicated, AEDs are a worthy tool to guide and control the quality of CPR-performance with real-time feed-back systems [2, 8]. Even more certain parameters like the depth and the frequency of chest compressions, the no-flow-time and the time until a shock is delivered can be captured, calculated and documented by some AEDs. Without the technical support of such systems these parameters from real CPR-scenarios are hard to obtain and erroneous in clinical routine.

In 2007 we started an AED-program at the Wuerzburg University Hospital (Germany). At the same time an AED- and CPR-training program for nurses was initiated. Although recent literature suggest that the lower survival rates of in-hospital cardiac arrest are associated with AED use [9, 10], little is known about the role of an AED in the CPR-choreography, the specific handling of an AED in the in-hospital setting and the impact of the AED on global CPR-performance.

Aim of the presented study was to monitor the impact of Automated External Defibrillators on the management of in-hospital cardiac arrest events.

Parameters of interest were the general setting of the cardiac arrest, the interface-management between First-Responder-Team (FRT) and the Medical Emergency Team (MET) the specific handling of the AED, the CPR-performance, ROSC-rate and the user satisfaction with the AED device and the emergency trolley as well.

## Methods

### Setting

The presented evaluation was part of the quality management program of the Wuerzburg University Hospital and as such a retrospective data analysis.

The data evaluation has been granted an exemption from requiring ethics approval. The exemption was granted by the ethic committee of the Wuerzburg University.

The Wuerzburg University Hospital is a maximum care hospital with all medical specialties available. One thousand two hundred fifty-three beds are available for 53.000 patients each year.

The AED-program was started in 2007. In total 120 AEDs (AED Plus® Fa. Zoll Medical Deutschland GmbH, Koeln, Germany) were installed at different locations all over the hospital (regular wards, diagnostic and therapeutic units, recovery rooms etc.). Simultaneous to the installation of the AEDs, the emergency trolleys and the emergency equipment were standardized and a CPR-training-program for nursing-staff was initiated. During the first year AED instructors gave the training to the nurses. Those nurses who were trained by AED instructors became trainers themselves and were then able to offer a qualified training to the rest of the nursing staff. To coach the trainers there is a teaching nurse, who has instructor qualification. The contents of the training were basic and advanced cardiac life support, handling and application of the AED.

Each AED was placed on a new and standardized emergency trolley so that a total of 120 emergency units were available all over the campus. ICUs, operating theatres and emergency rooms were not equipped with these emergency units because full-scale defibrillators and other emergency equipment are always available on scene and the staff of these areas is well trained in Advanced Cardiac Life Support. Focus of the training program, was AED-training, Basic Life Support and correct use of the emergency equipment. The medical emergency management plan of the Wuerzburg University hospital is based on a central resuscitation team. It consists of an anaesthesiologist and an ICU-nurse, which both have special training in intensive care and emergency medicine. The team is stationed on the ICU of the department of anaesthesiology and will be on scene within a maximum of 8–10 min after the emergency call. While the different medical departments are dispersed all over the campus an ambulance service secures the transfer of the emergency team to the emergency scene. The team is equipped with a full scale defibrillator (M-Series, Fa. Zoll Medical Deutschland GmbH, Koeln, Germany) and additional devices needed for Advanced Cardiac Live Support and difficult airway management.

### Data acquisition

The data acquisition was part of a continuous quality improvement process and thus part of the hospitals general quality management program. All obtained data consisted of routinely captured parameters during CPR by the AED. This data was anonymous and could not be correlated to a specific patient. It consisted of depth and frequency of chest compression, the no-flow-fraction, the initial rhythm and the time elapsed until the first compression started and until a shock was delivered when indicated. Chest compression data were captured via CPR-D.padz® with the Real CPR-Help®-technology (Fa. Zoll Medical Deutschland GmbH).

Every AED-use was reported by the first responder on scene to the medical head of the AED-program via e-mail. A questionnaire was completed by the first responder and was also sent to the medical head of the AED-program. The content of questionnaire is shown in Table 1. The resuscitation data was transferred from the AED to a personal computer via infrared interface and was stored anonymous in a data bank. The data of the questionnaire was linked anonymous to the data of the AED.

**Table 1:** Contents of the questionnaire

How was the emergency detected?	
Observed?	
Unobserved?	
Detected by patients monitor?	
CPR interruption in order to call the MET?	
Yes	
No	
Take Over FRT/MET	
ROSC?	
Continuation of CPR?	
Continuation of AED-use?	
Change to full-scale-defibrillator?	
Change of AED electrode-paddles?	
Outcome?	
ROSC	
Transfer to ICU under CPR	
Death on scene	
User satisfaction with the AED (Grades 1–6 (1 = best))?	
General Handling (Grades 1–6 (1 = best))	
Quality of the online feed back system (Grades 1–6 (1 = best))	
General value of the AED in the CPR-setting (Grades 1–6 (1 = best))	
User satisfaction with the emergency trolley (Grades 1–6 (1 = best))?	
Equipement (Grades 1–6 (1 = best))	
Clarity (Grades 1–6 (1 = best))	
Accesability (Grades 1–6 (1 = best))	

*MET* Medical Emergency Team, *FRT* First Responder Team, *ROSC* Return of Spontaneous Circulation, *AED* Automated External Defibrillator, *ICU* Intensive Care Unit, *CPR* Cardio Pulmonary Resuscitation

### Data analysis

For analysing the CPR parameters, the chest compression rate, compression depth, the no-flow-fraction, time interval to the first compression, the time interval to the first shock and the time interval to the first compression after the shock were calculated by Code Net Ventral Software (Fa. Zoll Medical Deutschland GmbH, Koeln, Germany). Consistent to the guidelines correct chest compression depth was defined as between 5 and 6 cm, correct chest compression rate was defined as between 100 and 120 per minute.

### Inclusion criteria

Every CPR-event with AED application that was reported to the medical head of the AED-program was included.

**Fig. 1:**
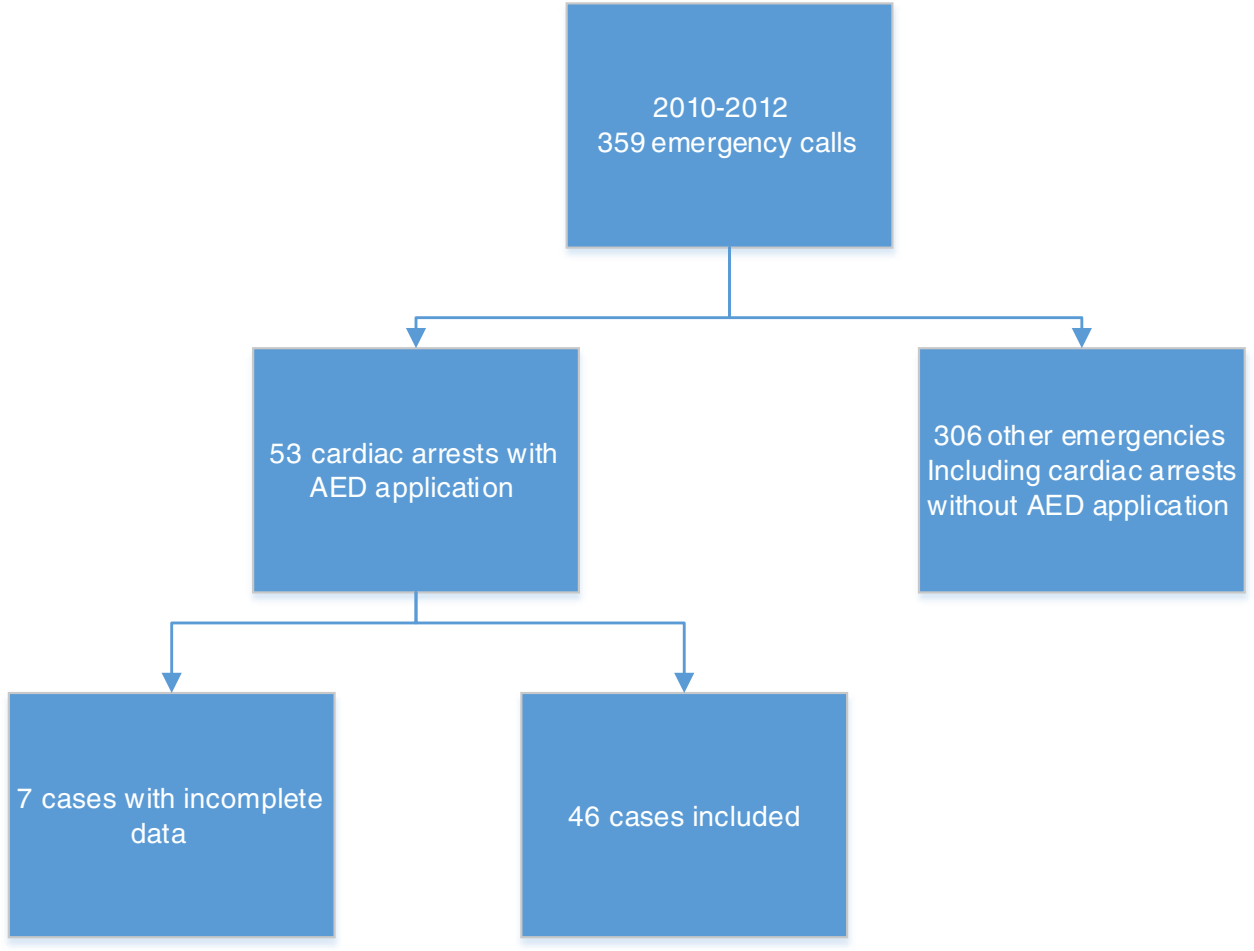
Exclusion process

The Exclusion Process is shown in Fig. 1.

### Outcome parameters

Quality of CPR-Performance:

- Chest compression rate (CCR)
- Depth of chest compression (CCD)
- No flow fraction (NFF) – defined as the quotient of the time without chest compression and the total time of cardiac arrest.
- Time interval from AED-activation to first compression (TtC)
- Time interval from AED-activation to first shock (TtS)
- Post shock pause (time interval to the first compression after a shock was delivered, TtCS)
- ROSC on scene
- Transfer to ICU under ongoing CPR
- Death on scene

Subjective Parameters requested by the questionnaire (expressed in german scholar-grades 1 = excellent to 6 = insufficient :

- Quality of the online feed back system
- Handling of the AED
- The general value of the AED in the CPR-setting
- Quality of the emergency trolley

### Statistics

Descriptive data was expressed as means and standard deviations for continuous variables. Time intervals were expressed as median with Interquartile range (IQR 25–75). Microsoft Excel for Windows (Version 2003, Microsoft Corporation, Redmond, USA) and SPSS (SPSS Inc, Il, USA) for windows 15.1 and 17.1 was used to analyse the data. AED data were calculated by Code Net Central Software (Fa. Zoll Medical Deutschland GmbH, Koeln, Germany).

## Results

From 2010 to 2012 the MET of the University Hospital Wuerzburg had to response to a total of 359 emergency calls. Out of these 53 were cardiac arrests with an AED-application. Complete data was available in 46 cases.

In 28 cases (61 %) the cardiac arrest was observed (6 cases were detected by a vital sign monitor a general ward). In 16 cases (35 %) the cardiac arrest was unobserved and in 2 cases (4 %) the general setting was unclear. In all cases (100 %) the first responders were able to make the emergency call without interruption of ongoing CPR.

A change from AED to a full-scale defibrillator was made in 29 cases (63 %) by the MET, a continuous use of the AED was found in 17 cases (37 %). In 6 cases the electrode-paddles of the AED were removed and replaced by the full-scale defibrillators electrode although this is not required since the AED-paddles are compatible with the full scale defibrillator.

The time intervals and NFF are shown in Table 2. The primary rhythms are shown in Table 3. In 15 out of 46 cases a shock was delivered. In 5 cases the time to the first shock was excluded from the analysis. In those cases a shock was delivered later in the scenario while the initial analysis showed a non-shockable rhythm.

**Table 2:** Time intervals and no flow fraction

Time interval from AED-activation to the first compression detected by the AED (TtC) (*n* = 40) (Median and IQR)	34 (32–52) seconds
No flow fraction (NFF) (*n* = 40)	41 %
Time interval from AED-activation to the first shock (TtS) (*n* = 10) (Median and IQR)	30 (28–32) seconds
Time from shock to the first compression after shock (TtCS) (*n* = 10) (Median and IQR)	4 (3–6) seconds

IQR = Inter Quartile Range

**Table 3:** Frequencies of primary rhythms

Asystole	*n* = 16
Ventricular Fibrillation	*n* = 7
Ventricular Tachycardia	*n* = 3
Pulseless Electrical Activity	*n* = 3
Bradycardia (<30 beats/min)	*n* = 8
Pulseless Tachycardia (>150 beats/min)	*n* = 5
Others	*n* = 4

In 6 out of 46 cases the CPR-parameters (CCD, CCR and NFF) were excluded from the analysis. In 3 cases there was successful defibrillation with ROSC after the first shock and only a very short period of chest compression was performed. In three other cases the CPR duration was less than two cycles. The depth of the chest compressions (*n* = 40) (mean ± standard deviation) was 5.5 ± 1 cm while the frequency of chest compressions (*n* = 40) (mean ± standard deviation) was 107 ± 11/min.

Return of spontaneous circulation (ROSC) was achieved in 9 cases (20 %) before the MET was on scene. Three of them had Ventricular fibrillation and a shock was delivered. In 37 cases (80 %) there was an ongoing CPR at the time of the MET arrival.

**Fig. 2:**
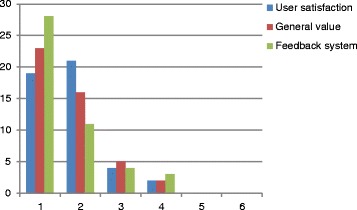
User evaluation of the AED (grades from German scholar system; 1 = excellent, 6 = insufficient)

**Fig. 3:**
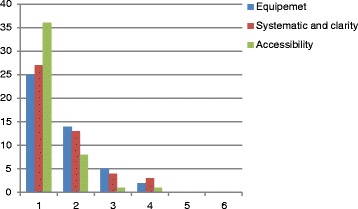
User evaluation of the Emergency Trolley (grades from German scholar system; 1 = excellent, 6 = insufficient)

ROSC was achieved in 21 patients (45 %), 8 patients (17 %) died on scene and 17 patients (37 %) were transferred under ongoing CPR to an Intensive Care Unit (ICU). User satisfaction with the AED, the general value and the quality of the AED’s feedback system is shown in Fig. 2. The grading of the Emergency Equipment, the systematic and the clarity of the emergency trolley is shown in Fig. 3.

## Discussion

By analysing AED-captured CPR-parameters in combination with the results of a questionnaire we were able to evaluate and analyse the initial setting of in-hospital cardiac arrest events, the handover management (FRT to MET), specific handling of the AED, CPR-performance, outcome and user satisfaction with the AED device and the emergency trolley.

Especially the handling of the AED and its incorporation in the CPR-choreography is an important issue and becomes more and more part of critical discussion [11–13]. The main question remains whether the use of an AED prolongs the hands-off time in CPR [14]. In our study we found a time elapse from switching on the AED to the first compression registered by the AED of 34 (32–52) seconds . The time from starting the AED to the delivery of the first shock was 30 (28–32) seconds . This indicates that the most users of the AED completely followed the voice prompts of the AED, being in detail: “stay calm”, “check for response”, “shout for help”, “stay calm”, “check for response”, “shout for help”, “open airway”, “check for breathing”, and”install the soft paddles on the patients undressed chest”. The “chain of advices” takes about 35 s. After all, as soon as the electrode-paddles are placed correctly on the patient’s chest the AED starts analysing the heart rhythm. This analysis takes about 10 s. The total delay can be markedly reduced, when the user is trained to interrupt the “chain of advices” by placing the electrode-paddles immediately on the patient’s thorax causing the AED to switch directly into the analysing mode. Our results suggest, that the “chain of advices” has not been interrupted by the AED users and that this might be associated with a prolongation of the TtS or even the NFF. Intensive training and adaption of training contents is needed to optimize the handling of the AED in order to maximize its advantages and to minimize its disadvantages.

Mueller et al. demonstrated a considerable variation in NFF, perishock pause and time to first shock among different commercially available AEDs [15]. While using eight different AEDs in simulated scenarios of cardiac arrest the time to the first chest compression was 50 ± 3 s. In these cases the “chain of advices” was not interrupted and the reported results correspond to our findings. In a second scenario the “chain of advices” was interrupted and the time to the first chest compression was significantly shorter and consequently the NFF was significantly reduced as well [15]. This strongly supports our hypothesis, that specific training is necessary to optimize the handling of the AED-device. While this is possible for in-hospital settings (training of medical staff) specific training of laypersons in the use of Public Access AEDs is much more difficult.

Fleischhackl et al. assessed important operational outcomes (the time elapse to the first shock and start of BLS) in 6 different AEDs. They included lay volunteers who were untrained in BLS and AED-use. The authors found significant differences between the 6 AEDs while the minimum time elapse to the first shock was 78 s and the maximum time was 128 s. The authors stated that factors of failure may have been related to the design of AED-hardware and the content and volume of the voice prompts [16]. This also corresponds to the interpretation of our results.

Comparing the user satisfaction with the AED (Fig. 2) with the objective performance (expansion of TtC and TtS while following the voice prompts), it becomes obvious that there was a lack of sense for the target to keep the TtC, TtS and the no-flow time as short as possible during CPR.

This is important to understand in order to optimize CPR-choreography, team training and training contents. Nolan et al. highlighted the importance of team training and choreography in order to provide high qualitative CPR [1]. In a consensus statement of the American Heart Association Meany et al. stated that the choreography of team activities is very important in order to maximize the cardiac compression fraction (CCF) [2]. They stated that any task that could be accomplished during ongoing chest compressions should be performed without introducing a pause [2]. The interruption of the “chain of advices” in order to shorten the TtS our findings is one important component to optimize the entire CPR-performance.

Looking at the proceedings when the FRT handed over the patient to the MET we found a change from AED to the full-scale defibrillator in 63 % and a continuous use of the AED in 37 % of the cases. In 6 cases the electrode-paddles of the AED were removed and replaced by the full scale defibrillators electrode-paddles. There is some data, that the take over by Advanced Life Support trained ambulance paramedics from rescuers using an AED is associated with shock delay and this is associated with decreased survival [17]. Our data show, that there is no consistent approach by the MET. In order to optimize CPR-choreography a standard approach is beneficial. This approach needs to be defined and integrated into the curriculum of team trainings. It is well known that the use of real-time feed back systems in combination with team training is associated with better resuscitation performance [18–20]. In case there is a change form AED to full-scale defibrillator it is advantagous when the full-scale defibrillator is equipped with real-time feed-back technology.

To determine the best practice (change to full-scale defibrillator or continuous use of AED) with regard to NFF and CPR-Choreography further research is needed. In our setting the change of the electrode-paddles is definitively superfluous and should be avoided.

CCR and CCD in our survey met the guideline recommendations. This result might be due to training and the use of a feedback-system. While there is no control group the evidence for this assumption is low.

In the presented study we found ROSC in 45 % of the patients, 8 patients (17 %) died on scene and 17 patients (37 %) were transferred under ongoing CPR to an Intensive Care Unit (ICU). In three patients (7 %) the AED delivered a shock and ROSC was achieved before the MET was on scene. We note that in these cases the AED was potentially lifesaving.

Smith et al. found a ROSC-rate of 54 % in 84 patients that had in-hospital cardiac arrest with AED-application, while patients without an AED-application had ROSC in 35 % [10]. Nolan et al. found a ROSC rate longer than 20 min in 45 % [21], which is comparable to our findings. In this study there was no information weather or not an AED was in use. Thus further interpretation of this study with regard to the influence of an AED is not possible. In a prospective study Müller et al. found similar ROSC-rates in patients after in-hospital cardiac arrest with and without the use of an AED [14]. They concluded that this could be due to an increase of the hands-off time while using an AED. In the same study the authors found an increasing ROSC-rate over time (2008–2012) in patients after in-hospital cardiac arrest from 48 % (2008) to 72 % (2012) [14]. One possible reason for this increase was a consequent training in BLS for nurses and physicians. This strongly supports our presumption, that training and AED-specific training contents are necessary to optimize CPR-performance and outcome.

### Limitations

The major limitation of our study is, that we did not determine the long time survival and neurological outcome of the treated patients. This is due to the initial conception of the study: Data drafted from the AED and the information from the questionnaire was anonymous and a correlation to a specific patient is not possible.

Another major limitation is the lack of a control group.

A further drawback is that we did not register those events in which an AED should have been applied but was not. This might cause a certain bias while the FRT possibly performed worse than in the study sample. On the other hand the focus of the observation was the quality of CPR while using an AED. Without AED application we were unable to monitor CCD, CCR, NFF, TtC, TtS and TtCS) and therefore even if those scenarios were registered, it would have been impossible to asses CPR-quality without AED data.

## Conclusion

AEDs might play an important role in the choreography of in-hospital CPR, especially as real-time feed-back systems. The total time elapse possibly can be reduced, when the user is trained to interrupt the “chain of advices” by placing the electrode-paddles immediately on the patient’s thorax. Our results suggest, that the “chain of advices” has not been interrupted by the AED users and that this might be associated with a prolongation of the TtS/TtC or even the no-flow time.

Intensive training and continuous monitoring and adaption of the training contents are needed to optimize the handling of the AED in order to maximize its advantages and to minimize its disadvantages.

## Abbreviations

AED: Automated external defibrillator
CCD: Depth of chest compression
CCR: Chest compression rate
CPR: Cardio-pulmonary resuscitation
MET: Medical emergency team
FRT: First responder team
NFF: No flow fraction
ROSC: Return of spontaneous circulation
TtC: Time interval to the first compression (after AED-activation)
TtS: Time interval to the first shock (after AED-activation)
TtCS: Post shock pause (Time interval to the first compression after a shock was delivered)
